# Body weight status and cardiovascular risk factors in adults by frequency of candy consumption

**DOI:** 10.1186/1475-2891-12-53

**Published:** 2013-04-30

**Authors:** Mary M Murphy, Leila M Barraj, Xiaoyu Bi, Nicolas Stettler

**Affiliations:** 1 Exponent®, Inc., Center for Chemical Regulation & Food Safety, Washington, DC 20036, USA

**Keywords:** Candy, Confectionery, NHANES, Adults, Obesity, Cardiovascular risk factors

## Abstract

**Background:**

Limited information is available regarding the impact of candy consumption on health. The purpose of this study was to investigate associations between typical frequency of candy consumption and body weight status and select cardiovascular risk factors among adults in the United States.

**Methods:**

Using data collected in the 2003–2006 National Health and Nutrition Examination Surveys (NHANES), adults were categorized as infrequent (≤ 3 eating occasions [EO]/month), moderate (> 3 EO/month and ≤ 3.5 EO/week), or frequent (> 3.5 EO/week) candy consumers based on the combined frequency of chocolate and other candy consumption over the previous 12 months. Weight and adiposity status were analyzed using logistic regression models, and blood pressure, lipids, and insulin sensitivity were analyzed using linear regression models. Models were adjusted for age, sex and race/ethnicity, and also for additional covariates with potential associations with the outcomes. Appropriate statistical weights were used to yield results generalizable to the US population.

**Results:**

Frequency of candy consumption was not associated with the risk of obesity, overweight/obesity, elevated waist circumference, elevated skinfold thickness, blood pressure, low density lipoprotein (LDL) or high density lipoprotein (HDL) cholesterol, triglycerides, or insulin resistance. Increased frequency of candy consumption was associated with higher energy intakes and higher energy adjusted intakes of carbohydrates, total sugars and added sugars, total fat, saturated fatty acids and monounsaturated fatty acids (p < 0.05), and lower adjusted intakes of protein and cholesterol (p < 0.001).

**Conclusions:**

Increased frequency of candy consumption among adults in the United States was not associated with objective measures of adiposity or select cardiovascular risk factors, despite associated dietary differences. Given the cross-sectional study design, however, it cannot be concluded that candy consumption does not cause obesity or untoward levels of cardiovascular risk markers. The lack of an association between frequency of candy consumption and cardiovascular risk factors could be due to reduced intake of candy among the overweight due to dieting or a health professional’s recommendations. Additionally, it is important to note that the analysis was based on frequency of candy consumption and not amount of candy consumed. Longitudinal studies are needed to confirm the lack of associations between frequency of candy consumption and cardiovascular risk factors.

## Background

The 2010 Dietary Guidelines for Americans encourage consumption of a nutrient dense diet, with reduced intakes of sodium, solid fats, added sugars, and refined grains [1]. Calories from solid fats and added sugars combined account for approximately 35% of total energy intakes in the US, or approximately 768 kilocalories per day [2]. Americans are encouraged to reduce consumption of both added sugars and solid fats as foods containing concentrated sources of these components are calorically dense foods that tend to replace nutrient-dense components of the diet and contribute to excess energy intake [1]. Because chocolate candy is a source of added sugars and saturated fat, and non-chocolate candy is a source of added sugars, the broad category of candy is often regarded as one of the first foods that must be limited or avoided to reduce consumption of added sugars and solid fats. However, the extent to which candy contributes to obesity and its main public health complications, diabetes and cardiovascular disease, is unclear and has recently been questioned [3-5].

The American Heart Association (AHA) recently issued recommendations for adults to limit added sugars intake to 100–150 calories per day, which corresponds to approximately half the total discretionary calorie allowance for most adults [6]. The association of added sugars intake with obesity is controversial [6-9]. Higher total intakes of added sugars have, however, been associated with increased triglycerides and decreased high density lipoprotein (HDL) cholesterol, and, in women, increased low density lipoprotein (LDL) cholesterol [7]. A strong body of evidence supports an association between higher intakes of saturated fat and increased risk of dyslipidemia [1]. Higher intakes of saturated fat compared to monounsaturated fat also have been associated with decreased insulin sensitivity [10,11]. In contrast to adverse effects on health associated with increased intakes of added sugars and saturated fat, evidence suggests that consumption of cocoa, a component of chocolate candy, may be associated with beneficial effects on cardiovascular risk factors [12-15].

While candy consumption, based on one day dietary recall data, appears not to be associated with an increased weight status and related cardiovascular risk factors [4,5], we are unaware of studies in which these associations were examined based on typical candy consumption over an extended period of time, which may be more relevant. Therefore, the purpose of this study was to investigate associations between typical frequency of candy consumption and body weight status and select cardiovascular risk factors among adults in the United States.

## Methods

### Data source and sample population

Data collected as part of the National Health and Nutrition Examination Surveys (NHANES) conducted in 2003–2004 and 2005–2006 were used to complete this cross-sectional study. NHANES is a continuous survey based on a complex multistage probability sample designed to provide nationally representative nutrition and health data and prevalence estimates for nutrition and health status measures in the United States [16,17]. Approval for NHANES data collection was provided by the National Center for Health Statistics Research Ethics Review Board. The current study was limited to adults age 19 years or above, excluding pregnant and lactating females, with food frequency questionnaire (FFQ) responses to questions on candy consumption and two complete 24-hour dietary recalls.

### Frequency of candy consumption (primary exposure)

In NHANES 2003–2004 and 2005–2006, a FFQ component was included to gather information on the frequency of foods consumed over the previous 12 months [18]. More recent releases of NHANES did not include the FFQ component and therefore could not be used for this analysis. The FFQ was developed by the National Cancer Institute (NCI) and was based on the NCI Diet History Questionnaire [19]. The 151 items on the FFQ represent a slight modification of the original NCI instrument. Portion size information was not collected with the FFQ. Printed FFQ questionnaires were mailed to the homes of English or Spanish-speaking survey participants 2 years of age and older who provided at least one complete 24-hour dietary recall interview.

The FFQ included two questions on candy consumption over the previous 12 months, namely: (1) how often did you eat chocolate candy; and (2) how often did you eat other candy. Definitions of chocolate and other candy were not provided with the FFQ, and the FFQ did not include a specific question regarding chewing gum. Based on their own interpretation of the two candy categories, survey respondents reported their typical frequency of consumption of each of the two types of candy as one of eleven specified frequency categories ranging from “never” to “2 or more times per day”. The FFQ data files were processed by the National Center for Health Statistics (NCHS) using the Diet*Calc software to provide daily frequencies of consumption, i.e., eating occasions per day (EO/d) by candy type for each respondent based on the categorical response. The Diet*Calc algorithm converted the highest frequency category of “2 or more times per day” to 2 EO/day. Previous studies [20] have found that subjects who eat some foods most frequently tend to eat more of these foods when they consume them, and consequently for some foods there is a positive correlation between the frequency of consumption and the amount of food consumed per eating occasion. The association between gram per eating occasion (g/EO) (from 24-hour recalls) and number of EO/day (from the FFQ) by type of candy consumed (chocolate and non-chocolate) was examined in a preliminary analysis using linear regression analysis for the subset of individuals who reported consumption of candy on one or both of the two days of dietary recall. Portion size of candy by eating occasion (g/EO) was not associated with the frequency of candy consumption in linear regression models of chocolate candy g/EO versus frequency of chocolate candy consumption and non-chocolate candy g/EO versus frequency of other candy consumption (R^2^ < 1%; data not shown). Adults who reported consuming one candy type in the previous 12 months were more likely to report consumption of the other candy type in the previous 12 months (Pearson chi-square test, p-value < 0.001), and the frequency of chocolate candy consumption was significantly associated with the frequency of non-chocolate candy consumption (p-value < 0.001, linear regression). Frequency of candy consumption as reported was therefore assumed to be representative of total candy intake. Previous assessments in adults have not shown negative effects on cardiovascular risk factors by type of candy [4,5], therefore daily frequencies of chocolate and other candy were combined into one variable for the present study to derive an estimate of daily frequency of total candy consumption. Based on summed responses in the FFQ for typical frequency of chocolate and non-chocolate candy consumption, adults were divided into three groups based on typical frequency of candy consumption:The ranges within each of the frequency categories were subjectively selected to provide roughly similar proportions of the population in each group while also corresponding to easily interpretable frequencies of consumption over the course of a month or week.

•Infrequent: ≤ 3 EO/month (daily frequency ≤ 0.09 eating occasions per day);

•Moderate: > 3 EO/month and ≤ 3.5 EO/week (daily frequency > 0.09 and ≤ 0.5 eating occasions per day); or

•Frequent: > 3.5 EO/week (daily frequency > 0.5 eating occasions per day).

### Physiologic parameters (primary outcomes)

During the examination component of NHANES, participants underwent a physical examination. As part of the examination, body measurement data were collected by trained health technicians following NHANES anthropometry protocols [21]. Body mass index (BMI, kg/m^2^) was calculated as body weight divided by height squared. Skinfold calipers were used to measure sub-scapular and triceps skinfolds. Sub-scapular and triceps skinfold measurements coded in the data release as “exceeds capacity” (i.e., the amount of adipose tissue exceeded the limits of the caliper) were assigned values of 44 and 45 mm, respectively, as these values correspond to the highest reported value by type of measurement. The sub-scapular and triceps skinfold measurements were then summed per individual. Approximately 5% of the summed values included an assigned maximum value.

Up to three measurements each of systolic and diastolic blood pressure were taken from subjects after resting quietly in a sitting position for 5 minutes [22]. Fasting or non-fasting blood samples were collected for lipid profile and insulin levels. Triglycerides, glucose and insulin were measured only in individuals randomly selected to participate in the morning session after an overnight fast. Triglycerides were measured enzymatically in serum using a series of coupled reactions in which triglycerides are hydrolyzed to produce glycerol [23]. Total and HDL cholesterol were measured in samples from all individuals. HDL cholesterol was measured colorimetrically after precipitation of the apoB containing lipoproteins [24]. LDL cholesterol levels provided in the data release were calculated using the Friedewald calculation: [LDL-cholesterol] = [total cholesterol] – [HDL-cholesterol] – [triglycerides/5] (if triglycerides was 400 or less), where all values are expressed in mg/dL [23]. Plasma fasting glucose was determined by a hexokinase method and insulin was measured with an immunoassay method [25]. For subjects with fasting plasma glucose and serum insulin data, we calculated insulin sensitivity using the quantitative insulin sensitivity check index (QUICKI), defined as 1/[log(I_o_) + log(G_o_)] [26].

### Demographics and other participant characteristics

Demographic characteristics including age, sex, race (categorized as non-Hispanic white, non-Hispanic black, Mexican American, other Hispanic, or other), and education level (categorized as less than high school graduate, high school graduate/GED or equivalent, or beyond high school) were self-reported by participants [27]. The family poverty income ratio (PIR) was derived from family income data; the data were released as a continuous variable, with all PIRs of 5 or more coded as 5.

The in-home questionnaire included separate questions about participation in vigorous physical activity and participation in moderate physical activity over the previous 30 days for at least 10 minutes; participants provided a yes or no response to each question [28]. For this analysis, adults were categorized into one of three physical activity groups based on the highest level of activity reported: none, moderate or vigorous. Participants also were asked to quantify the average number of hours per day of sitting and watching television (TV) or videos during the previous 30 days; responses were coded as 0 (representing anything less than 1 h), or 1, 2, 3, 4, or 5 or more hours/day [28]. Self-reported use of medication was captured during the in-home questionnaire including use of insulin or diabetic pills for control of diabetes, medication for elevated blood pressure, and medication to lower cholesterol levels [22,29]. Cotinine concentrations were derived in serum samples collected from survey participants [30]. For this analysis, participants with blood cotinine levels of ≥ 3 ng/mL were classified as smokers [31]. Demographic and other lifestyle characteristic information was used as covariates in analyses of some parameters in the current study.

### Nutrient intakes

The dietary interview component of NHANES is known as “What We Eat in America” (WWEIA) [32,33]. In this component of the survey, trained dietary interviewers collected detailed information on all foods and beverages consumed by respondents in the previous 24 hour time period (midnight to midnight). A second dietary recall was administered by telephone 3 to 10 days after the first dietary interview, but not on the same day of the week as the first interview. In the current study, nutrient concentration data in the United States Department of Agriculture (USDA) Food and Nutrient Database for Dietary Studies (FNDDS) version 3.0 [34] were used to calculate nutrient intakes by respondents in the combined NHANES 2003–2006. The MyPyramid Equivalents Database (MPED) 2.0 was the source of added sugars concentration data and total fruit and vegetable servings [35]; values for foods consumed in NHANES 2005–2006 but not included in MPED were imputed based on values for similar foods or recipe calculations. Total fruit and vegetable intakes, total intakes of select nutrients, and non-candy intakes of select nutrients were used as covariates in some analyses. Non-candy nutrient intakes were calculated as the difference between total 2-day average nutrient intakes and 2-day average nutrient intakes from foods within the candy category (excluding gum) of the USDA hierarchical food coding scheme as presented in FNDDS [34].

### Statistical analysis

Descriptive characteristics including age, sex, race/ethnicity, education, PIR, percent participation in physical activity in the previous 30 days, average daily time sitting and watching TV or videos, and smoking status were summarized by frequency of candy consumption category. Ordered logistic regression models with category of frequency of candy consumption as outcome and each of the descriptive characteristics as predictors were used to compare subjects in the three candy consumption categories.

Estimates of usual nutrient intake (from the 24-hour dietary recalls) were derived using the approach developed by Nusser et al. [36] and Carriquiry [37]. Software for Intake Distribution Estimation (C-Side; version 1.02, 1997, Iowa State University Statistical Laboratory, Ames, IA) which implements this method was used to estimate the usual nutrient intakes. Estimates of usual intakes of energy, protein, fat, saturated fat, total sugars, added sugars, and fiber were generated for subpopulations of adults by frequency of typical candy intake. It was not possible to obtain usual intakes of alcohol using the C-Side model as the model is designed to halt if it fails to result in an acceptable semi-parametric normality transformation. Hence, estimates of 2-day average intakes of alcohol were developed and used in the analyses. Energy adjusted nutrient intakes were determined using the residual method [38].

Obesity was defined as a BMI of 30.0 or higher, and overweight or obese was defined as a BMI of 25.0 or higher. Waist circumference of 102 cm or higher in men and 88 cm or higher in women was defined as elevated based on the defined risk factors for metabolic syndrome [39]. Summed skinfold thickness values at or above the 95th percentile of the age- and sex-specific reference values compiled from the first and second National Health and Nutrition Surveys were considered to represent excessive skinfold thickness [40].

Weight and adiposity status were analyzed using logistic regression with the log of the odds of being obese, overweight/obese, or having an elevated waist circumference or skinfold thickness as outcome variables, with indicator variables for belonging to categories of candy consumption frequency as predictors. Two models were run. The first included non-modifiable confounding factors, namely sex, age and race/ethnicity (model 1), while the second model included these factors as well as categorical factors for education, income, smoking status, physical activity and time watching TV/videos (model 2). Ordered logistic regression models with category of candy consumption frequency as the outcome were used to conduct tests for trend in both models.

Blood pressure, lipids, and insulin sensitivity were analyzed using linear regression models with the physical measures as outcome variables, with indicators for category of candy consumption frequency as predictors. Two models were run. The first included non-modifiable confounding factors, namely sex, age and race/ethnicity (model 1) while the other model also included other variables likely to be associated with the physical measures, including categorical factors for education, income, smoking status, physical activity, time watching TV/videos and whether taking blood pressure, cholesterol or diabetes medication (model 2); and continuous variables for BMI and energy-adjusted dietary factors associated with cardiovascular disease risk [41,42]. Because blood pressure, cholesterol or diabetes medications have an impact on blood pressure, lipid profile, and insulin sensitivity, they were introduced into the models as dummy variables. This allowed the analysis to take into account the variability of cardiovascular risk factors in all subjects, including those who were on medication and to investigate the potential association of candy consumption with blood pressure, lipid, and insulin sensitivity control for these subjects. Ordered logistic regression models with category of candy consumption frequency as the outcome were used to conduct tests for trend in both models.

The tests were conducted using STATA (StataCorp. 2007, Stata Statistical Software: Release 10, College Station, TX: StataCorp LP) and used the statistical weights developed by NCHS to adjust for the differential probability of selection and non-response and to adjust for the complex statistical design of NHANES. The sampling weight produced by NCHS for FFQ respondents (WTS_FFQ) was used in analyses of demographic characteristics, nutrient intakes, anthropometrics, blood pressure, and HDL cholesterol. Because only a subset of NHANES participants was examined in the morning session and fasted, special sampling weights (referred to as the “fasting” weight) were also generated by NCHS to appropriately analyze outcomes assessed in this subsample. The fasting sampling weight was used in analyses of LDL cholesterol, triglycerides, plasma glucose and insulin.

## Results

### Characteristics of adults by frequency of candy consumption

**Figure 1:**
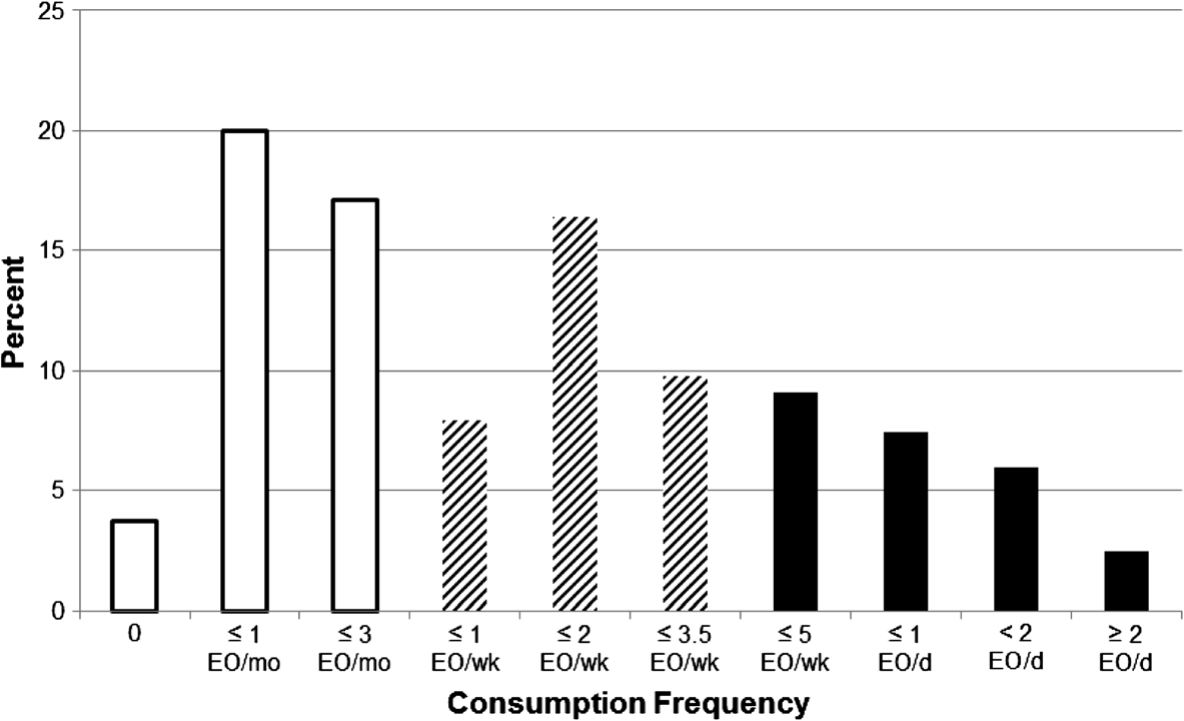
**Distribution of frequency of total candy consumption among adults 19+ y, NHANES 2003–2006.** EO: eating occasions. White bars represent infrequent consumption of candy; hashed bars represent moderate frequency of consumption, and solid bars represent frequent consumption.

The sample population included the 5817 adults age 19 years and older with FFQ responses to questions on candy consumption and two complete 24-hour dietary recalls. The distribution of typical frequency of candy consumption among adults is presented in Figure 1. Among adults, 41% of the population was classified as infrequent candy consumers; approximately 10% of this population (or 4% of all adults) reported never consuming candy in the previous year. Two percent (2%) of adults reported eating candy on two or more occasions per day. The majority of all adults reported consuming chocolate candy (93%) or other candy (87%) at least once in the past twelve months, and 84% reported consuming both chocolate and other candy in the previous twelve months (data not shown). Among all adults, 65% reported consuming candy on no more than two occasions per week (data not shown).

Descriptive characteristics of the population by frequency of candy consumption category are summarized in Table 1. As shown in Table 1, increased frequency of candy consumption was associated with younger age, a higher proportion of non-Hispanic whites, and lower proportions of Mexican Americans, non-Hispanic blacks, and adults categorized as “other race” (p < 0.05). More frequent consumption of candy also was associated with a lower proportion of adults with less than a high school education and a higher proportion with education beyond high school, and higher income as measured by the PIR (p < 0.05). Levels of physical activity, time spent watching TV or videos, and smoking status did not differ by frequency of candy consumption.

**Table 1:** Characteristics of adults 19+ y by frequency of candy consumption, NHANES 2003-2006

	**Frequency of candy consumption**			
	**≤ 3 EO/month**	**> 3 EO/month and ≤ 3.5 EO/week**	**> 3.5 EO/week**	
	**(Infrequent)**	**(Moderate)**	**(Frequent)**	
**Characteristic**		**Mean ± Standard error**		**P for trend**
Population, n (%)	2497 (41)	1954 (34)	1366 (25)	**-**
Age, y	48 ± 0.6	46 ± 0 .6	46 ± 0.6	0.003
Gender,% male	48 ± 1.0	45 ± 1.73	45 ±1.69	0.125
Race/Ethnicity,%				
Mexican American	8 ± 1.0	7 ± 1.35	6 ± 1.03	0.021
Other Hispanic	3 ± 0.6	2 ± 0.39	2 ± 0.46	0.129
Non-Hispanic White	69 ± 2.7	76 ± 2.21	80 ± 2.47	< 0.0005
Non-Hispanic Black	13 ±1.6	11 ± 1.36	9 ± 1.50	0.004
Other Race	7 ± 0.9	4 ± 0.6	3 ± 0.8	< 0.0005
Education,% completing^a^				
Less than high school	18 ± 1.2	14 ± 0.8	12 ± 1.1	< 0.0005
High school/GED	25 ± 1.2	30 ± 1.6	27 ± 2.4	0.130
More than high school	57 ± 1.6	56 ± 1.8	61 ± 2.5	0.037
Poverty Income Ratio (PIR)	3.0 ± 0.06	3.1 ± 0.06	3.2 ± 0.08	0.010
Physical Activity,% reporting in previous 30 days^a^				
None	33 ± 1.5	30 ± 1.7	31 ± 1.7	0.136
Moderate	32 ± 1.2	35 ± 1.4	33 ± 1.5	0.649
Vigorous	35 ± 2.0	36 ± 1.7	37 ± 2.0	0.401
Watching TV/videos, h/d^a^	2.3 ± 0.05	2.3 ± 0.05	2.4 ± 0.07	0.192
Smoking status,% yes^a^	31 ± 1.5	28 ± 1.9	28 ± 1.8	0.118
Usual macronutrient intake, nutrient/d^b^				
Energy, kcal	2101 ± 17.1	2192 ± 24.0	2311 ± 27.8	< 0.0005
Protein, g	85 ± 0.4	83 ± 0.4	79 ± 0.7	< 0.0005
Carbohydrate, g	259 ± 1.4	263 ± 1.4	271 ± 1.8	< 0.0005
Total sugars, g	116 ± 1.1	121 ± 1.4	130 ± 1.6	< 0.0005
Added sugars, g	74 ± 1.4	80 ± 1.5	88 ± 1.7	< 0.0005
Dietary fiber, g	15.9 ± 0.22	16.0 ± 0.19	15.6 ± 0.21	0.385
Total fat, g	82.7 ± 0.44	84.8 ± 0.57	84.0 ± 0.52	0.015
Saturated fat, g	27.3 ± 0.22	27.9 ± 0.18	28.0 ± 0.22	0.014
Monounsaturated fat, g	30.4 ± 0.16	31.3 ± 0.24	31.2 ± 0.19	<0.0005
Polyunsaturated fat, g	17.8 ± 0.11	18.5 ± 0.17	18.0 ± 0.21	0.181
Cholesterol, mg	298 ± 3.5	279 ± 3.0	265 ± 3.3	< 0.0005
Alcohol, g	21 ± 1.2	15 ± 1.2	15 ± 1.4	0.003

^a^ Estimates based on available data; data missing for some subjects.

^b^ Nutrient intakes adjusted for energy intake.

Energy and macronutrient intakes by frequency of candy consumption also are shown in Table 1. Increased frequency of candy consumption was associated with higher energy intakes and higher energy adjusted intakes of carbohydrates, total sugars and added sugars, total fat, saturated fatty acids and monounsaturated fatty acids (p < 0.05). Increased frequency of candy consumption was associated with lower energy adjusted intakes of protein and cholesterol (p < 0.001), and intake of alcohol (p < 0.05). Energy adjusted intakes of fiber and polyunsaturated fatty acids did not differ across the candy frequency categories.

### Anthropometrics and physiological parameters and candy consumption

Frequency of candy consumption was not associated with the risk of obesity, overweight/obesity, elevated waist circumference, elevated skinfold thickness, blood pressure, LDL and HDL cholesterol, triglycerides and insulin resistance in models adjusted for age, sex and race/ethnicity (Model 1) or in the model with adjustments for additional covariates (Model 2) (Tables 2 and 3, respectively). Additionally, frequency of candy consumption was not associated with BMI when analyzed as a continuous variable rather than as a categorical variable as presented in Table 2 (data not shown).

**Table 2:** Odds ratios (OR) of elevated weight and adiposity status in adults 19+ y by frequency of candy consumption, NHANES 2003-2006

**Body measure**	**Frequency of candy consumption**												**P for trend**
	**≤ 3 EO/month**				**> 3 EO/month and ≤ 3.5 EO/week**				**> 3.5 EO/week**				
	**(Infrequent)**				**(Moderate)**				**(Frequent)**				
	**n**	**OR**	**95% CI**	**p-value**	**n**	**OR**	**95% CI**	**p-value**	**n**	**OR**	**95% CI**	**p-value**	
% obese													
Model 1^a^	2456	1.00	- -	- -	1925	1.04	(0.84, 1.27)	0.720	1343	1.01	(0.8, 1.26)	0.954	0.880
Model 2^b^	2251	1.00	- -	- -	1787	1.02	(0.83, 1.24)	0.866	1220	1.01	(0.81, 1.27)	0.907	0.855
% overweight/obese													
Model 1^a^	2456	1.00	- -	- -	1925	1.06	(0.9, 1.25)	0.490	1343	0.89	(0.76, 1.05)	0.153	0.319
Model 2^b^	2251	1.00	- -	- -	1787	1.00	(0.84, 1.18)	0.966	1220	0.89	(0.75, 1.05)	0.162	0.262
% with high waist circumference^c^													
Model 1^a^	2413	1.00	- -	- -	1893	0.97	(0.81, 1.18)	0.777	1316	0.96	(0.8, 1.16)	0.674	0.697
Model 2^b^	2227	1.00	- -	- -	1762	0.95	(0.79, 1.14)	0.566	1203	0.98	(0.82, 1.16)	0.799	0.741
% with skinfold thickness ≥95th percentile^d^													
Model 1^a^	1901	1.00	- -	- -	1497	0.80	(0.49, 1.32)	0.375	1048	0.92	(0.56, 1.5)	0.733	0.612
Model 2^b^	1745	1.00	- -	- -	1391	0.87	(0.54, 1.41)	0.559	956	0.96	(0.55, 1.68)	0.874	0.736

^a^ Adjusted for sex, age, and race/ethnicity.

^b^ Adjusted for sex, age, race/ethnicity, education, PIR, smoking (Y/N), physical activity, and time watching TV/videos.

^c^ High waist circumference defined as ≥102 cm for men and ≥88 cm for women [39].

^d^ 95th percentile of subscapular and triceps skinfold [40].

**Table 3:** Blood pressure, lipids, and insulin sensitivity in adults 19+ y by frequency of candy consumption, NHANES 2003-2006

	**≤ 3 EO/mo**			**> 3 EO/mo and ≤ 3.5 EO/wk**			**> 3.5 EO/wk**			**P for trend**
	**(Infrequent)**			**(Moderate)**			**(Frequent)**			
	**n**	**mean** ± **SE**	**p-value**	**n**	**mean** ± **SE**	**p-value**	**n**	**mean** ± **SE**	**p-value**	
Systolic blood pressure										
Model 1^a^	2388	123 ± 0.5	reference	1882	124 ± 0.4	0.257	1312	124 ± 0.9	0.496	0.411
Model 2^b^	2169	123 ± 0.5	- -	1731	123 ± 0.4	0.393	1181	124 ± 0.9	0.286	0.237
Diastolic blood pressure										
Model 1^a^	2372	71 ± 0.4	- -	1866	71 ± 0.3	0.540	1304	72 ± 0.5	0.107	0.112
Model 2^b^	2153	71 ± 0.4	- -	1716	71 ± 0.3	0.360	1174	72 ± 0.5	0.424	0.557
LDL cholesterol										
Model 1^a^	1023	115 ± 1.2	- -	827	117 ± 1.3	0.304	595	118 ± 2.4	0.290	0.249
Model 2^c^	965	116 ± 1.3	- -	800	116 ± 1.3	0.733	558	118 ± 2.5	0.399	0.395
HDL cholesterol										
Model 1^a^	2403	55 ± 0.5	- -	1877	54 ± 0.5	0.563	1299	54 ± 0.6	0.864	0.811
Model 2^d^	2250	54 ± 0.5	- -	1783	54 ± 0.5	0.937	1218	54 ± 0.5	0.778	0.740
Triglycerides										
Model 1^a^	1049	143 ± 4.0	- -	847	137 ± 3.5	0.250	608	145 ± 5.4	0.815	0.957
Model 2^e^	978	144 ± 4.1	- -	808	141 ± 4.0	0.472	560	149 ± 6.1	0.499	0.578
Insulin sensitivity (QUICKI)										
Model 1^a^	1047	0.34 ± 0.002	- -	846	0.35 ± 0.002	0.249	605	0.35 ± 0.002	0.168	0.111
Model 2^f^	976	0.34 ± 0.001	- -	807	0.35 ± 0.002	0.161	557	0.35 ± 0.002	0.258	0.131

^a^ Adjusted for sex, age, and race/ethnicity.

^b^ Adjusted for sex, age, race/ethnicity, education, PIR, smoking (Y/N), physical activity, time watching TV/videos, BMI, blood pressure medication (Y/N), and energy adjusted alcohol, sodium, potassium, calcium, and total fruit and vegetable intake.

^c^ Adjusted for sex, age, race/ethnicity, education, PIR, smoking (Y/N), physical activity, time watching TV/videos, and cholesterol medication (Y/N), and energy adjusted alcohol, non-candy fiber, non-candy saturated fat, non-candy monounsaturated fat, non-candy polyunsaturated fat, and non-candy cholesterol intake.

^d^ Adjusted for sex, age, race/ethnicity, education, PIR, smoking (Y/N), physical activity, time watching TV/videos, BMI, cholesterol medication (Y/N), and energy adjusted alcohol intake.

^e^ Adjusted for sex, age, race/ethnicity, education, PIR, smoking (Y/N), physical activity, time watching TV/videos, BMI, cholesterol medication (Y/N), and energy adjusted alcohol and non-candy total sugars intake.

^f^ Adjusted for sex, age, race/ethnicity, education, PIR, smoking (Y/N), physical activity, time watching TV/videos, BMI, diabetes medication (Y/N), and energy adjusted alcohol intake.

## Discussion

In contrast to what is often assumed, the present study showed that increased frequency of candy consumption was not associated with obesity or cardiovascular risk factors including blood pressure, lipid profile, and insulin sensitivity.

Adults with higher frequency of candy consumption consumed diets higher in energy, energy-adjusted carbohydrates, total sugars, added sugars, total fat, and saturated and monounsaturated fatty acids, and diets lower in protein, cholesterol and alcohol. Frequency of candy consumption was not, however, associated with objective measures of adiposity including BMI, waist circumference and skinfold measures, or with objectively measured cardiovascular risk factors including blood pressure, HDL or LDL cholesterol, or insulin sensitivity. No association was observed between frequency of candy consumption and the adiposity and cardiovascular parameters in models adjusted only for age, sex and race/ethnicity or in models adjusted for additional covariates with potential associations with the outcomes, including socioeconomic status (education, income), physical activity and sedentary activity, other dietary components including nutrients provided by foods other than candy, smoking, weight status, and use of medications for health conditions including elevated blood pressure, elevated cholesterol, and diabetes. The 95% confidence intervals (CI) of the odds ratio (OR) and the differences in point estimates of continuous variable combined with the standard error (SE) suggest that large undetected differences between groups or type 2 errors are unlikely.

The absence of any association between frequency of candy consumption and measures of adiposity or cardiovascular risk factors despite the fact that candy is a source of added sugars and saturated fat may in part be due to the relatively minor contribution of candy consumption to these dietary components. Based on NHANES data used in our assessment, adults were estimated to consume an average of 44 kilocalories (kcal) daily from candy [43]. Candy accounted for slightly more than one teaspoon of added sugars (approximately 5 g) or 20 kcal in the diets of adults on a daily basis [44], which corresponds to a small fraction of the 100–150 calorie prudent upper limit of added sugars recommended by the AHA [6]. Similarly, based on NHANES 2007–2008, Welsh and colleagues recently reported that candy and gum provided 4.5 to 6.4 g of added sugars in the diets of adults [45]. Candy accounted for 3.1% of the total saturated fat intake by the US population aged 2 years and older in 2005–2006, or slightly less than 1 g based on a total saturated fat intake of 27.8 g/day [46,47].

In contrast to the relatively modest contributions of candy to added sugars and saturated fat intakes, the top three dietary sources of added sugars for adults – sugary drinks, grain-based desserts, and sweetened fruit drinks – account for approximately 60% of the total added sugars intake [44]. Sugary drinks alone, including sodas, energy drinks, sports drinks and fruit drinks, provide approximately 9.4 teaspoons of added sugars intake among adults, or approximately eight times the average amount provided by candy [44]. Grain-based desserts provide approximately 2.8 teaspoons of added sugars in the diets of adults, or slightly more than twice the amount provided by candy, while dairy desserts provide approximately 1.2 teaspoons of added sugars, which is comparable to the amount provided by candy [44]. The dietary sources of saturated fat are more diverse than the sources of added sugars. Cheese is the top ranked source of saturated fat in the US diet, accounting for 8.5% of total saturated fat intake, followed by pizza (5.9%), of which the saturated fat is presumably attributable primarily to cheese and a lesser extent to meat toppings [46].

Another possible reason that candy consumption was not found to be associated with unfavorable cardiovascular risk factors may be that although candy is a relatively minor component of the diet, cocoa-containing candy specifically can be a significant source of flavanols [48]. Flavanols have been associated with beneficial effects on cardiovascular risk factors [12-14]. Additionally, stearic acid accounts for approximately one third of the total fat in cocoa butter and the majority of cocoa butter’s saturated fat [49]. Unlike other saturated fatty acids, stearic acid is not known to raise LDL cholesterol levels [50]. The lack of an association between frequency of candy consumption and cardiovascular risk factors could also be due to reverse causality, namely individuals identified with cardiovascular risk factors may be advised by their health care professionals to limit intake of saturated fat and added sugars, including candy. This possibility cannot be excluded in a cross-sectional study such as the present one when the outcome (e.g., bodyweight status) causes people to behave differently with respect to the exposure, namely candy intake.

As reporting of energy intake in dietary assessment tools is mostly dependent on body size, physical activity and under-reporting, the observed differences in energy intake between groups are not necessarily suggesting a more positive energy balance in frequent consumers of candy. Additionally, associations between frequency of candy consumption and usual macronutrient intakes cannot be attributed solely to candy. In order to better understand diet quality across the three categories of candy consumption, we conducted a post-hoc assessment of Healthy Eating Index-2005 (HEI-2005) scores [51] by frequency of candy consumption. Mean HEI-2005 scores of infrequent, moderate, and frequent candy consumers were 59.9 (95% CI: 58.0, 61.7), 57.5 (95% CI: 55.9, 59.2), and 56.7 (95% CI: 54.7, 58.7), respectively; the scores were not significantly different from one another, and are comparable to the mean HEI-2005 score of 57.2 for all adults in the US [52]. Therefore, despite the observed associations between macronutrient intakes and frequency of candy consumption, diet quality as assessed by a comprehensive measure was not associated with frequency of candy intake. Although dietary factors have strong relationships with weight and cardiovascular risk factors, individual genetic and non-nutritional lifestyle factors also contribute to these factors. Furthermore, although the differences in intake of added sugars and saturated fat between groups were statistically significant, due to the large sample size, these differences may be too small to have a clinically significant impact on obesity and cardiovascular outcomes.

In the current analysis, adults were categorized into one of three categories based on reported frequency of total candy consumption during the past 12 months in a FFQ, which could be more relevant to health outcomes that develop over long periods of time than intakes based on a one- or two-day diet recall. Over the course of a year, nearly all adults - approximately 96% - reported consuming candy at least once. Use of the FFQ in this analysis therefore allowed for discrimination of adults into infrequent, moderate and frequent categories of consumption based on candy consumption over an extended period of time, thus avoiding the potential for misclassification of adults as non-consumers of candy based on just one day of recall.

The three frequency categories used in the analysis distinguish infrequent candy consumers from those who typically consume candy on most days and those with typical frequency of candy consumption between the two extremes. Given the range of reported frequency of candy consumption in the top category, there is heterogeneity in consumption within this group and potentially also outcomes. In order to better understand associations between the most frequent candy consumers and measures of body weight status and cardiovascular risk factors, a post-hoc analysis was conducted in which adults consuming candy more than once per day (8% of adults) were compared to all other adults. Results from this analysis showed that the most frequent candy consumers were not significantly more likely to be overweight or to have more adverse measures on cardiovascular risk factors (data not shown). These findings may appear to be somewhat unexpected, though are not entirely surprising in that some overweight individuals may eat candy less frequently due to dieting or in response to a healthcare professional’s guidance, and consequently underscore the challenges of using cross-sectional data to study diet and health associations. Additionally, given that portion size data were not collected in the FFQ, we do not know if the most frequent candy consumers in fact ate the most candy. Furthermore, for most people who consume candy more than once per day, the contribution of candy to the overall diet is likely still small and may be insufficient to have a meaningful health impact.

There is limited information in the literature on associations between candy consumption and measures of body weight and cardiovascular risk factors among adults. In a cross-sectional assessment based on a one-day dietary recall, O’Neil and colleagues [4] found that body weight, BMI, waist circumference, and risk of elevated diastolic blood pressure were lower in adults who reported consumption of candy compared to those who did not. Analyses by type of candy showed lower body weight and waist circumference in chocolate candy consumers compared to nonconsumers, and in sugar candy consumers compared to nonconsumers; mean BMI was lower in adults who reported consumption of sugar candy compared to those who did not, and consumers of chocolate candy had a reduced risk of lower HDL cholesterol and metabolic syndrome compared to nonconsumers of chocolate [4]. In contrast to the current study, however, the study by O’Neil and colleagues was based on one day of dietary recall data rather than typical frequency of candy consumption, which could be more relevant to health outcomes that develop over long periods of time; approximately 22% of adults were identified as candy consumers in that study. In a cross-sectional assessment of adult males, median BMI was slightly though significantly higher among candy consumers compared to men classified as non-consumers (24.41 kg/m^2^, interquartile range (IQR): 22.95-26.44 and 24.39 kg/m^2^, IQR: 22.69-26.22, respectively, p < 0.001) [53]. More recently, findings from a cross-sectional assessment of 1018 healthy adults showed that greater weekly frequency of chocolate consumption was associated with lower BMI (beta coefficient = −0.208, SE = 0.06, p = 0.001 in adjusted model) [3].

This study is a cross-sectional study, and therefore causality cannot be determined. Because of this limitation, longitudinal studies of associations between typical consumption of candy and anthropometric and physiologic measures are needed to better understand the role of candy in measures of health among adults. If the absence of associations between anthropometrics and physiologic measures observed in the current study is confirmed in longitudinal studies, the findings may help to focus concerns on dietary components more strongly associated with obesity and cardiovascular disease risk.

Other limitations of the study must also be considered. As with all dietary surveys, the accuracy of the estimates derived from reported intakes is limited by the accuracy of responses provided by survey participants. Misreporting of dietary intakes, specifically under-reporting of energy, occurs with both FFQs and 24-hour dietary recalls, and is more likely among obese than normal-weight individuals [54-56]. An analysis of data collected in 24-hour dietary recalls indicated that candy is among the food groups less likely to be reported by low energy reporters, and when reported, reported less frequently and in smaller portions [57]. Reported frequencies of candy consumption on the FFQ may additionally be limited by self-interpretation of what constitutes “candy” as definitions of chocolate and non-chocolate candy were not provided. For example, individuals may or may not have interpreted non-chocolate candy to include gum or mints, and chocolate covered confections may have been categorized as chocolate or non-chocolate candies. Additionally, it was not possible to classify adults based on typical amount of candy consumed given that portion size information was not collected in the FFQ. Also, it is important to note that the intent of this analysis was to identify associations between frequency of consumption of all types of candy and the selected measures of health. If different types of candy have different effects on health, this analysis would reflect only the net effect.

There are, however, several strengths to the present study. The analysis was based on a large, nationally representative sample of the US population and classification of frequency of candy consumption was derived from reported typical consumption patterns over the past year using a tested instrument, providing a measurement of candy consumption more relevant to health and less susceptible to misclassification than measurements based on a single or two-day recall. All anthropometric and physiologic measurements were collected following established protocols.

## Conclusion

In conclusion, results from this study indicate that increased frequency of candy consumption among adults in the United States is not associated with objective measures of adiposity including BMI, waist circumference and skinfold measures, or cardiovascular risk factors including blood pressure, HDL or LDL cholesterol, or insulin sensitivity as assessed by the QUICKI indicator. Previous research has shown that for the average adult, candy is a relatively small source of energy, added sugars or saturated fat. Within the ranges of candy frequency reported by adults and assessed in this study, it is possible that the amount of added sugars and saturated fat consumed through candy may be too small relative to other dietary, lifestyles, or genetic factors to have a measurable effect on health outcomes. Longitudinal studies are needed to confirm associations between frequency of candy consumption and cardiovascular risk factors.

## Abbreviations

AHA: American Heart Association; BMI: Body mass index; C-Side: Software for Intake Distribution Estimation; CI: Confidence interval; EO: Eating occasion; FFQ: Food frequency questionnaire; FNDDS: Food and Nutrient Database for Dietary Studies; HDL: High density lipoprotein; HEI: Healthy Eating Index; IQR: Interquartile range; kcal: Kilocalories; LDL: low density lipoprotein; MPED: MyPyramid Equivalents Database; NCHS: National Center for Health Statistics; NCI: National Cancer Institute; NHANES: National Health and Nutrition Examination Survey; OR: Odds ratio; PIR: Poverty income ratio; QUICKI: Quantitative insulin sensitivity check index; SE: Standard error; TV: Television; US: United States; USDA: US Department of Agriculture; WWEIA: What We Eat in America; Y/N: yes or no.

## Competing interests

The authors declare that they have no competing interests.

## Authors’ contributions

MMM, LMB and NS designed the study. MMM, XB and LMB analyzed the data; LMB provided statistical expertise. MMM, LMB and NS interpreted the study, drafted the manuscript, and critically revised the manuscript for important intellectual content. XB provided administrative and technical support throughout the study. All authors read and approved the final manuscript.
